# Case of a Variety of Intermittent Fever, Accompanied by Symptoms of Hydrophobia, and Forming an Intermittent Hydrophobic Fever, (Febris Hydrophobica)

**Published:** 1803-11-01

**Authors:** C. L. Dumas

**Affiliations:** Member of the National Institute, President of the Society, &c.


CASE *of a* VARIETY *of* INTERMITTENT FEVER, *accompanied by Symptoms of* HYDROPHOBIA, *and forming an* INTERMITTENT HYDROPHOBIC FEVER, (Febris Hydrophobica); *read at the Medical Society of Montpelier, by* C. L. DUMAS, *Member of the National Institute, President of the Society, &c.*

THE moderns, observes the author, have improved no part of the practice of physic so much as our knowledge of intermittent or dangerous remittent fevers. This order of diseases offers the best proofs that can be brought to establish the certainty and resources of the medical art; here the best answer is found to the detractors of a science, which has so many titles to the first rank amongst useful inventions. If the physician was ever worthy of being compared to God, it is doubtless when calculating the unavoidable dangers of a remittent fever left to itself, he can announce the precise moment when it shall terminate either favourably or otherwise. The probability of death following a certain number of paroxysms of this fever; that of a cure succeeding the timely and judicious administration of bark, are facts founded on the united testimony of such multiplied observation as to amount to unquestionable certainty. In enumerating the symptoms and dangerous consequences of intermittent fevers, Torti remarks those only which he had most frequently met with. The dangerous symptoms which this celebrated practitioner enumerated are cholera morbus, dysentery, bilious discharges, sweats, syncope, long continued shivering, and lethargy. Hence the fever was particularly characterised according to the predominant symptom; such as, the choleric, hepatic, dysenteric fever, &c. Mercatus had already noticed many of these species under the head of varieties of the dangerous tertian fever; all the rest he comprehended under the title of fevers, which became dangerous from some foreign cause, (pernitiosæ ob aliquod accedens quod lucerescit); he adds too, other circumstances of difference which Morton has elegantly marked. Torti, who did not think necessary to speak of appearances which he had not seen, does not however exclude them. All fevers which become dangerous from the association of some symptom are tacitly comprised in the first division, and agreeably to him should be treated according to the same method. (Omnes nihilominus quotquot sunt que vitæ periulum inferunt ex quòvis adjuncto symptomate tacite comprehensas eademque simul methodo curandas.) Among the symptoms which Morton has noticed connected with intermittent fevers, we principally find those of pleurisy, peripneumony, cephalalgia, rheumatism, &c. Lautter has described fevers accompanied by epilepsy. Galiazzi gives instances of fever attended by all the symptoms of asthma. Torti speaks of a double tertian accompanied by delirium. Alibert, speaking of irregular intermittents, notices very accurately those fevers which have delirium for a leading symptom. Delirium, observes the author, is an affection which takes on a great variety of characters. A horror of liquids, and a wish to bite whatever comes near, are modifications which are sometimes observed; such instances are however uncommon, though they must have been met with in practice. A kind of symptomatic madness, which comes on in certain cases of malignant or putrid fever, and which frequently accompanies inflammation of the stomach and angina, has been already remarked. Boerhaave gives the case of a man who attended criminals to the place of execution, and was suddenly attacked with fever, of which he died at the end of three days, and during which he drove from him with horror every kind of fluid. Salius Diversus mentions the case of a spontaneous hydrophobia which develloped itself in a woman of 36 years of age, at the conclusion of pestilential fever, and during which the hydrophobic symptom was so remarkable, that she could not suffer a person to drink in her presence. Hippocrates mentions a case of fever fever where this symptom was predominant; and similar instances are to be found in Koehler, Massa, Hoffman, Sauvages, Mead, James, and many others. Vogel speaks of hydrophobia when treating of fevers, and says that it may spontaneously occur in the course of high inflammatory fevers. Selle says that spontaneous hydrophobia may occur in the course of many fevers, particularly of the nervous kind. There can be no doubt, observes M. Dumas, but that the same symptom may occur in the course of intermittent fevers; but as yet, facts are wanting to prove the truth of this association. The only one within the author's knowledge is given by Lentieri, in the case of an old man, who after exposing himself to cold was attacked with a remittent fever of a malignant kind, accompanied by delirium and a horror of water, but which disappeared on the use of bark. Nor has Lentieri mentioned all the details necessary to characterize accurately the *febris hydrophobica remittens.* M. Dumas having found no instance of this kind in any medical work, considers himself entitled to the credit of being the first to give an account of this particular fever, with all its attendant circumstances.

In the year 1793, the author had occasion of observing a fever of this kind in the Civil Hospital of Lyons, and since which period he has constantly mentioned it in his medical lectures; and Dr. Alibert, on his testimony, has reckoned it among the varieties of irregular intermittents in a dissertation which he has published on the subject, but it has been no where described as in the history of the following case.

A man, forty-five years of age, of a dry temperament, nervous, iritable, melancholic, a heated imagination, and violent character, and who had for a long time given himself up to singular irregularities of regimen, was obliged to pass many nights in a tent exposed to the firing of cannon, and the explosion of bombs; laying himself down on damp ground he fell asleep, but was soon awakened by a sense of cold and general uneasiness. The following day he experienced some giddiness, accompanied with pain in the head and every unpleasant sensation throughout his members. At night he had a shivering fit, followed by slight heat, obscure vision, loss of appetite, and prostration of strength. These symptoms appeared on the 26th of August, 1793.

27th. The patient was free of any similar sensations, except that he complained of cephalalgia, and continual vomiting of greenish matter, which fatigued him much. The pulse and heat of the body were natural.

28th. At night a shivering came on, succeeded by a stronger stronger degree of heat than after the preceding attack, accompanied by ardent thirst, a fixed pain about the throat, difficulty in swallowing, and slight delirium. The patient was ordered an emulsion with nitre, and after the conclusion of this access he was brought to the hospital.

29th. M. Dumas found the remission of the fever complete. He appeared however in a state of stupor and dejection, and complained much of a tightness in the muscles of the neck; at night there was a slight degree of fever but unaccompanied by either cold or sweat; the pulse retained a degree of irregularity sufficient to excite mistrust; an emulsion with nitre and a clyster were the only remedies used this day.

30th. There was a very decided access of fever, and the characters of a tertian intermittent were manifest; the paroxysm was marked by some disagreeable appearances, and it exceeded the former both in strength and duration. The heat was considerable, the delirium furious, with convulsive motions in the muscles of the neck and lips, and deglutition became more difficult. The patient was violently agitated, threw off his bed clothes, struck his breast, and complained of the tightness of his throat, and which the impression of fluids increased; the tongue was dry with red edges, and blackish in the centre; and to judge from the motions of his members, one would have thought the patient already far advanced in an acute disease. The emulsion was continued as before, except with the addition of camphire; and fomentations of vinegar to the legs, with the application of four leeches to each ankle, were ordered. The access was prolonged to a late hour of the night, accompanied by the same symptoms throughout.

September 1. All the symptoms had disappeared, and were suceeded by a perfect calm; and with the exception of a singular dislike to drink, and a difficulty of swallowing, the patient experienced no inconvenience.

This sudden change from what was observed the preceding day engaged all the author's attention, and led him to suspect that he had something unusual to combat in the disease.

[ To be continued. ]

